# Case of Obstinate Constipation

**Published:** 1817-10

**Authors:** R. H. King


*For the London Medical and Physical Journal.*

*Case of obstinate Constipation;* by R. H. King, Esq.

WHETHER the successful termination of the following case was owing to the remedies, or to the efforts of Nature, it appears worthy of being recorded, as shewing to what an extent certain remedies may be safely employed, and encouraging us not to despair of recovery under circumstances apparently the most unfavourable.

Mrs. B., aged 40, a hard-working woman, and the mother of two children, had lost her father and two sisters from obstruction in the bowels, after very short illnesses. One of the latter was under my care, and her complaint was decidedly peritoneal inflammation. Mrs. B. is of a spare habit, naturally costive, but had been particularly so for a month previous to the present attack. On Wednesday, July 16th, she complained of considerable uneasiness in the bowels, in consequence of which, she took a dose of castor-oil, which produced three scanty motions, but without relieving her:—to use her own words, "it did not seem to have work'd off properly." She continued very much indisposed during the following day (the 17th), attending to her domestic concerns, till night, when she had a severe rigor, which was soon followed by acute pain round the umbilicus, and excessive vomiting. About two o'clock on Friday morning (18th), my assistant was sent for to her, the pain and sickness continuing. He immediately took sixteen ounces of blood from the arm, and sent her a pill containing hydrarg. submur. gr. vj. and a purging saline mixture to take after it. The mixture was rejected, and, the pain continuing tinuing without any evacuation from the bowels, about nine o'clock she took another pill, containing hydr. submur. gr. viij. and after it a full dose of castor-oil, which was directed to be repeated, if necessary, and if the stomach would bear it. The pain, however, continued, increasing at intervals; and the stomach rejected every thing. The pulse was about 80, not hard; the blood did not exhibit the least mark of inflammation; and the pain in the abdomen was not increased by pressure. During the night and the following day (19th) the sickness abated, the pains became less severe, and, for some moments, almost ceased entirely, when she passed two or three scanty motions, perfectly fluid, of a dark green colour, with particles floating in them resembling tea-leaves. From this time till early in the morning of the 27th, she had no evacuation whatever from the bowels, except about two table-spoonfuls of a matter resembling, both in colour and consistence, melted pitch, with scarcely any smell. During this period she had several intervals of ease, although, at times, the pain recurred with great severity, and she suffered greatly from nausea, vomiting, and occasionally hiccup. She was sometimes so extremely ill, with a low and fluttering pulse, sunken countenance, and cold extremities, that her death was almost looked upon as certain,—when she would again rally, the pulse becoming moderately full and soft, varying in frequency from 72 to 86, which latter it never exceeded. During the whole of her illness, the tongue was moist, very little furred, and she seldom or never complained of thirst. There was no fullness in any part of the abdomen, nor was the pain ever increased on pressure, till after she had swallowed the quicksilver, when, on strong pressure being made in the left hypochondrium, some degree of hardness might be felt, and it occasioned slight pain. During the first five or six days she passed very little urine, which was extremely high-coloured and turbid, resembling coffee-grounds. Almost every variety of purgative, and in every form, was tried, both by the mouth and in clysters, which last were thrown up with considerable force, in the quantity of five or six pints at once, and retained in the rectum as long as she could bear it; but they always returned very slightly discoloured, almost as soon as the pipe was withdrawn. Fomentations were applied to the abdomen, she went several times into the warm bath, and, on the 23d, the pain having returned with increased violence, and continued for several hours without the slightest intermission, she was again bled very freely, the blood exhibiting the same healthy appearance as before. Opiates were administered once or twice to procure sleep, and the tobacco clyster was tried, but occasioned such distressing sickness and languor that it was not deemed adviseable to repeat it. Every remedy having failed, my assistant proposed to try the effect of pure quicksilver, to which I consented, as, from the absence of inflammatory symptoms, I did not apprehend that it would do mischief, and, considering that it was probably a case of intus-susceptio, I thought that, if it happened to be in such a part of the intestinal canal as that the quicksilver might act favourably by its weight, it might possibly do good. At any rate, as the patient must have died if a passage could not be procured, I thought we were justified in trying it. Accordingly, on the afternoon of the 24th, she swallowed half a pound of quicksilver, which she said she felt run quickly through her, till it stopped at the part which had been the principal seat of pain. She felt little or no inconvenience after taking it, and, nothing having passed on the following morning (25th), she was directed to swallow another half pound, which she did, but without having the desired effect. For several hours in the forenoon of the 27th she felt a constant desire, and made several fruitless efforts, to go to stool. This entirely ceased about two o'clock, and in the evening I determined to try the effect of the ol. terebinth. rectif. of which she took six drachms made up in a draught with simple syrup and mucilage. This was about eight o'clock. Soon after taking the draught, she felt extremely sick, and vomited a little; and about midnight became so very faint and languid, that she was thought to be dying. This lasted for near an hour, when she called for assistance, and nearly half filled the vessel with very dark-coloured offensive slimy fœces, at the bottom of which was a part of the quicksilver, divided into very minute globules. She had several more stools in the course of the day, in all of which a few globules of the quicksilver appeared; but it was not till the evening that the smell of the turpentine could be perceived in the motions, when it became very strong. From this time the bowels have continued to act pretty freely, with the assistance of medicine; and on the 30th she voided at once 9½ oz. of quicksilver in one mass. Her stools have now acquired a perfectly healthy appearance, and she is in every respect convalescent, although very weak, having taken no sustenance for eleven days, except thin gruel, and that generally in small quantities, which were frequently rejected as soon as swallowed. Her strength has been still further reduced by a copious salivation, which came on in a day or two after the bowels began to act, probably from the quantity of calomel she had taken, which exceeded a drachm in the whole.

The

The quicksilver which appeared in the motions was unaltered; but, as the small quantities which sometimes passed could not be accurately collected and weighed, it is impossible to say whether the whole of it came away in its metallic form.

*Mortlake; Aug.* 13, 1817.

---

*For the London Medical and Physical Journal.*

